# Victims of medical errors and the problems they face: a prospective comparative study among the Dutch population

**DOI:** 10.1093/eurpub/ckaa106

**Published:** 2020-07-10

**Authors:** Peter G van der Velden, Carlo Contino, Arno J Akkermans, Marcel Das

**Affiliations:** c1 CentERdata, Tilburg University’s Network on Health and Labour (Nethlab), Tilburg, The Netherlands; c2 Fonds Slachtofferhulp (FSH), The Hague, The Netherlands; c3 Faculty of Law, Vrije Universiteit Amsterdam, Amsterdam, The Netherlands

## Abstract

**Background:**

A large number of studies are devoted to medical errors, but only a few focused on the problem’s victims of these errors face. Prospective comparative studies on this topic are absent. The aim of this prospective comparative study is to fill this gap of scientific knowledge that may help to improve the care for victims.

**Methods:**

Data were collected in the Longitudinal Internet studies for the Social Sciences panel, based on a random sample of the Dutch population. Surveys were conducted in March–April 2018 (T1^response^ = 82.1%) and March–April 2019 (T2^response^ = 80.1%). We assessed medical errors and potentially traumatic or stressful events between T1 and T2, and mental health, work, financial, religious, family, legal/administrative and physical problems at T1 and T2 (*N*^total^ = 4711).

**Results:**

In total, 79 respondents were affected by medical errors between T1 and T2, and 2828 were not affected by any event. Of the victims, 28% had high PTSD symptom levels at T2. Stepwise multivariate logistic regression entering all problems at T1 and demographics showed that victims compared with controls significantly more often had all assessed problems at T2, except family problems. For instance, victims more often had mental health problems (29.5% vs. 9.3%; adj. OR = 3.04, *P = *0.002) and financial problems (30.4% vs. 6.6%; adj. OR = 4.82, *P *<* *0.001) at T2.

**Conclusions:**

Victims of medical errors more often face various non-physical problems than others. Care for victims should therefore, besides physical health, also include the assessment and targeting of their problems regarding mental health, work, religion, legal issues and finance.

## Introduction

Medical errors are common and are responsible for considerable patient harm.^1^ The large majority of studies on medical errors focused on aspects of these errors such as, but not restricted to, the prevalence of these errors,^1^^,^^2^ the improvement in patient safety,^3^ patients’ need to be heard after medical errors^4^ and the negative impact of medical errors on health professionals and healthcare organizations.^5^

Remarkably, relatively few peer-reviewed cross-sectional studies focused on the *victims* of medical errors and the problems they face because of these errors. These studies have shown that individuals affected by medical errors, besides physical problems, may suffer from mental health, work, financial and legal problems in months, years and decennia after the medical error.^5–8^ However, to what extent individuals affected by medical errors are more at risk for these problems than non-affected persons is unknown. A literature search with PubMed and PsycInfo did not identify one peer-reviewed empirical comparative study on this topic. Although this may be due to the fact that awareness and disclosure of medical errors is on the rise.^9^

Moreover, prospective studies assessing the risk of these problems after medical errors while taking pre-event problems into account, are absent. Current studies on the effects of medical errors are all, for obvious reasons, conducted *after* the event. Therefore, it is unknown to what extent medical errors increase the risk of the aforementioned post-event problems. We do not know to what extent problems among victims of medical errors were already present before the medical error took place. However, insight in this risk is important for the development of care for these patients as well as for compensation schemes and redress. To fill this gap of scientific knowledge the present prospective comparative population-based study was conducted.

The research question was to what extent do adult individuals affected by medical errors more often have mental health, work, financial, partner/family, religious, legal/administrative problems and any non-physical problem than adults not affected by medical errors or other potentially traumatic of stressful life-events (SLE), while controlling for pre-event problems (including physical problems) and demographics.

## Methods

### Procedures and participants

This study is part of the longitudinal Victims in Modern Society (VICTIMS) study.^10^^,^^11^ This study builds upon the information provided by victims, patients and carers, although they were not directly involved in this study.^12^ Data were collected in the Dutch Longitudinal Internet studies for the Social Sciences panel. This panel is administered by CentERdata^13^ and funded by the Netherlands Organization for Scientific Research. It is based on a large representative random sample drawn from the Dutch population register by Statistics Netherlands (CBS). Panel members receive an incentive of 15 euro per hour and those who do not have a computer and/or internet access are provided with the equipment at home. All gave their informed consent. Data are available for scientific research, free of charge (see https://www.lissdata.nl, in English).

In March–April 2018 participants were administered the first survey of the VICTIMS study (T1, response = 82.1%). In total, 4711 of the 5879 adult respondents at T1 participated in the second survey in March–April 2019 (T2, response = 80.1%). The questionnaire was approved by a panel of external and internal reviewers of CentERdata. The first survey was weighted using 32 demographic profiles of the Dutch adult population (13.7 million), based on sex (males and females), age categories (18–34, 35–49, 50–64 and 65 years and older), marital status (married and not married) and employment status (employed and not employed) data of CBS (is 2 × 4×2 × 2 = 32 profiles). The results presented in the study are based on the total weighted sample. At T1 and T2, the number of missing values is very low (<0.05%) and the response at T2 was 80.1%. Because we cannot predict which participants will become victim of a medical error between T1 and T2, we did not impute our data. We have no information about why respondents did not participate at T1 or T2.

### Measures

At T2, respondents were asked whether they were victimized by a medical error (or medical accident) in the past 12 months (between T1 and T2), besides 20 other potentially traumatic events (PTEs) varying from serious threat, to (un)expected loss of a significant other (1 = no, 2 = yes), and one open question about possible PTEs not listed.^10^^,^^11^ For this study, we distinguished individuals affected by medical errors from respondents not affected by PTEs or SLE in the 12 months between T1 and T2.

In case respondents were confronted with two or more events, they were asked to take the most disturbing event in mind and rate the level of stress during the event (1 = not or almost, 2 = little, 3 = fairly, 4 = much and 5 = very much). For the present study these scores were recoded as very stressful (4 and 5) or not very stressful (1, 2 and 3). In addition, PTSD-symptomatology following this PTE was assessed using the 8-items version of the PCL-5^14–16^ that assessed symptoms across the four symptom clusters of PTSD according to DSM-5. Items have 5-point Likert scales and focus on symptoms in the past month (0 = not at all to 4 = extremely; Cronbach’s alpha=0.92). Scores were dichotomized in low and high event-related PTSD-symptom levels using a cut-off of 13.^17^

Problems at T1 and T2 were assessed with the brief Problems and Help Inventarisation-List (PHIL).^18^ Respondents were asked if they had physical problems, mental health problems, problems with religion, problems at work, problems in the family/with partner, financial problems and legal/administrative problems (1 = yes and 2 = no). The PHIL also assessed the use of professional help, but here we focus on problems only.

Mild-severe anxiety and depression symptoms were examined using the 5-item MHI-5 with 6-point Likert scales (6 = never to 1 = continuously).^19^ A cut-off of <60 was used to identify respondents with mild to severe anxiety and depression symptoms.^20^

### Analyses

Multivariate logistic regression analyses were conducted with problems at T2 as dependent variables. Predictors were medical errors between T1 and T2, the corresponding problem at T1 and all other problems at T1, sex, age, education level and employment status at T1, totalling 13 predictors. In our analyses, controls were treated as the reference category. To limit the number of predictors relative to the number of each problem at T2 as much as possible, e.g. to obtain higher events-per-variable (EPV) ratios,^21^^,^^22^ we used the stepwise procedure for entering the predictors in the logistic regression analyses with the following criteria: *P* values In (PIN) 0.05 and *P* values Out (POUT) 0.10.

For the prediction of work problems at T2, we excluded respondents of 65 years and older because of retirement. Respondents were considered to have ‘any’ non-physical problem at T2, if they suffered from mental health, religious, family, financial problems and/or legal/administrative problems. Analyses were conducted using IBM SPSS 25 using the weighted sample.

## Results

Of the total study sample (*N* = 4711), 79 respondents became victim of a medical error between T1 and T2 (1.7%, 95% confidence interval = 1.35–2.08). The control group of respondents not affected by any potentially traumatic or SLE consisted of 2828 adults (60.1%, 95% CI = 58.62–61.42). The demographic characteristics of the two subgroups are presented in table 1. Both groups differ significantly in age, employment status and marital status, but not in sex and highest achieved education level.

**Table 1:** Demographic characteristics of victims of medical errors (*N* = 79) and controls (*N* = 2828) at T1

	Victims medical errors (*N *=* *79), *N* (%)	Controls (*N *=* *2828),^a^ *N* (%)	*χ* ^2^ (*df*)	*P*
Gender				
Males	38 (48.1)	1421 (50.2)		
Females	41 (51.9)	1407 (49.8)	0.142 (1)	0.707
Age (years)				
18–34	27 (34.2)	750 (26.5)		
35–49	8 (10.1)	677 (23.9)		
50–64	18 (22.8)	711 (25.1)		
≥65	26 (32.9)	691 (24.4)	10.231 (3)	0.016
Highest education level				
Primary school^b^	16 (20.3)	656 (23.2)		
Higher secondary education^c^	1 (1.3)	182 (6.4)		
Intermediate vocational^d^	21 (26.6)	678 (24.0)		
Higher vocational^e^	24 (30.4)	777 (27.5)		
University	17 (21.5)	536 (19.8)	4.271 (4)	0.371
Employment status				
Employed	51 (65.4)	1367 (48.3)		
Unemployed	27 (34.6)	1461 (51.7)	8.828 (1)	0.003
Marital status				
Married	51 (65.4)	1451 (51.3)		
Unmarried	27 (34.6)	1377 (48.7)	6.023 (1)	0.014

Because of the weighting, cell counts have been rounded and therefore do not always exactly equal the sample size of both groups.

a Respondents not affected by any potentially traumatic or life-event in the past 12 months.

b Primary school, including intermediate secondary education, junior high school, not yet started any education and other.

c Higher secondary education/preparatory university education, senior high school.

d Intermediate vocational education, junior college.

e Higher vocational education, college.

For 66 of the 79 respondents affected by medical errors (83.5%), the medical error was the only/most disturbing event between T1 and T2. In total, 57.7% reported that they experienced the event as very stressful (much or very much) at the time of the event. At T2, 27.7% had high medical error-related PTSD symptom levels (for 13 respondents affected by medical errors, data about medical error-related stress and PTSD symptomatology are not available).

The prevalence of problems among respondents affected by medical errors and controls as well as the results of the stepwise logistic regression analyses are presented in table 2. Table 2 shows that victims of medical errors more often had problems regarding their mental health, work, finance, religion, legal/administrative issue and any non-physical problems at T2 than controls. All adjusted Odd Ratios for medical errors with controls as reference category were significant. For instance, 29.5% of the patients had mental health problems at T2, compared with 9.3% of the control group (adjusted OR = 3.04). To prevent lengthy tables, in table 2 we only showed the adjusted Odd Ratios for the predictor medical errors. The other predictors entered in the stepwise analyses are mentioned in table 2. The full tables predicting various problems at T2 are presented in Supplementary appendix S1.

**Table 2:** Summary results stepwise multivariate logistic regression analyses predicting problems at T2

	Victims medical errors (*N *=* *79), *N* (%)	Controls (*N *=* *2828),^a^ *N* (%)	aOR (95% CI)	*P*
Mental health problems at T2				
No	55 (70.5)	2566 (90.7)		
Yes	23 (29.5)	263 (9.3)	3.04 (1.50–6.16)^b^	0.002
Problems with work at T2^c^				
No	40 (76.9)	2002 (93.6)		
Yes	12 (23.1)	136 (6.4)	2.38 (1.12–5.05)^d^	0.021
Financial problems at T2				
No	55 (69.6)	2640 (93.4)		
Yes	24 (30.4)	188 (6.6)	4.82 (2.40–9.70)^e^	<0.001
Problems with religion at T2				
No	68 (86.1)	2798 (98.9)		
Yes	11 (13.9)	30 (1.1)	12.08 (5.10–28.64)^f^	<0.001
Problems with partner/family at T2				
No	66 (83.5)	2651 (93.7)		
Yes	13 (16.5)	178 (6.3)	Not entered^g^	NA
Legal/ administrative problems at T2				
No	67 (84.8)	2760 (97.6)		
Yes	12 (15.2)	68 (2.4)	4.00 (1.80–8.89)^h^	0.001
Any non-physical problem at T2				
No	35 (44.3)	2305 (81.5)		
Yes	44 (55.7)	523 (18.5)	5.08 (2.86–9.02)^i^	<0.001
Physical problems at T2				
No	25 (31.6)	1968 (69.6)		
Yes	54 (68.4)	860 (30.4)	4.93 (2.64–9.21)^j^	<0.001

aOR, adjusted odds ratio with controls (not affected by PTE or SLE) as reference category. All events -per-variable (EPV) ≥ 16.

a Respondents not affected by any potentially traumatic of life-event in the past 12 months.

b Adjusted for age; employment status; physical problems and mental health problems T1.

c For the prediction of problems at work, we excluded respondents of 65 years and older (*n* = 2190). All events-per variable (EPV) > 16.

d Adjusted for education level; physical, work and religious problems T1.

e Adjusted for age; employment and marital status and financial problems T1.

f Adjusted for problems religion T1.

g Education level, physical and family/partner problems T1 were entered, but not medical errors.

h Adjusted for mental health, family, financial and legal problems T1.

i Adjusted for age, gender, employment status, physical problems and ‘any’ problem T1.

j Adjusted for age, gender, employment status and physical health problems T1.

No significant difference was found for problems in family/with partner, since this variable was not entered in the analyses according to the PIN 0.05 criterion. Predicting mild-severe depression and anxiety symptoms according to the MHI-5 (not shown in table 2: see Supplementary appendix S1), instead of mental health problems according to the PHIL, revealed similar differences. Respondents who were affected by medical errors were more at risk for mild-severe depression and anxiety symptoms than controls (36.7 vs. 13.9%, adj. OR = 2.09, 95% CI = 1.13–3.87, *P* = 0.019). As could be expected, they also more often suffered from physical problems at T2 than controls (see table 2).

Of all respondents affected by medical errors, 28 (35.0%) reported no other PTE, 21 (27.0%) one other PTE and 29 (37.1%) two or more other PTEs. However, we found no indications that the last two subgroups had significant more problems than the first, while controlling for the corresponding problem at T1 and gender (we did not control for all other control variables because of the small sizes).

## Discussion

Of the total study sample, 1.7% became victim of a medical error between T1 and T2. Translated to, for example, the Dutch adult population, our results suggest that a considerable group of about at least 185 101 individuals (95% CI= 185 101–286 365) were affected by a medical error in a 1-year period. According to a report of the European Commission in the Netherlands 17% of the population had suffered themselves or had a family member that suffered from a serious medical error in a local hospital.^23^ With respect to a serious medical error from a medicine that was prescribed by a doctor, 9% reported that they or a family member had suffered from this type of medical error. These prevalence, based on surveys among the general population, are more of less comparable with countries such as the UK (18 and 11%), Spain (18 and 8%), and France (19 and 11%).^23^ The number of formal compensations claims in the Netherlands in the period 2007–16 varied between about 1400 and 1800 per year.^24^

Findings of the present prospective study showed, in line with the relatively few previous qualitative and quantitative cross-sectional studies,^5^^,^^7–9^ a clear pattern. Respondents affected by medical errors more often had problems regarding mental health, work, religion, finance and legal issues compared with non-affected adults, e.g. adults not affected by any of the assessed potential traumatic events or SLE. In the analyses this control group was treated as the reference group. No significant differences were found with respect to problems with the family or partner. Importantly, differences in the prevalence of problems between respondents affected by medical errors and non-affected controls cannot be attributed to pre-event problems, because in the stepwise multivariate logistic regression analyses existing problems were included and thus controlled for.

Nevertheless, replication of our prospective study using larger samples and especially studies in other countries is warranted. Comparison of outcomes across countries may shed lighter on to what extent individuals affected by medical errors are better off in certain health care and legal systems compared with other systems. Future research may help to identify which medical errors and which circumstances put individuals affected by medical errors more at risk for post-event problems than other individuals affected by medical errors. In addition, future prospective studies covering a longer post-event period are needed to examine the persistence of problems, given the results of the cross-sectional study of Ottosen *et al*.^7^ suggesting that even on the long-term (10 years or later after the event) victims suffer from various problems following medical errors.

Our study has strengths and limitations. A major strength is our study design: our study is based is based on a random sample of the Dutch population with pre-event assessments of problems regarding mental health, work, finance, family/partner, religion and legal issues among victims of medical errors and non-affected controls. Our findings were based on self-reports of respondents about the medical errors and the problems they face. Awareness of a medical error may be considered a multistage process. After an error has been committed (according to existing definitions), an individual must become aware of the event and define it as an error. However, in the tendency of the affected individual to define the event as an error the relationship with the caregiver may play a role. Unfortunately, we have no information about this relationship and the interactions between the affected respondent and caregiver, about how the caregiver evaluated the reported medical error and about which problems the social environment noted. Nevertheless, in the study of Zhu *et al*.^25^ more than 70% of the adverse events reported by patients were confirmed by physician reviewers. In addition, about 28% of the victims for whom data about medical error-related PTSD symptoms were available, had high or clinical PTSD symptom levels. Almost 60% experienced the event as very stressful suggesting that the reported events were serious. We used the PHIL^18^ to assess various problems at both surveys, but we have no information about the persistence of the problems on the longer term and about the provided support of involved medical or legal professionals, which is also of relevance for the development of care problems after medical errors.^8^ The response in our study was high at both T1 and T2 (>80%) but it possible in principle, although we consider that as highly unlikely, that panel members affected by medical errors without problems did not participate at T2 which could lead to an overestimation of problems among our sub sample of respondents affected by medical errors. The question about medical errors focused on medical errors in the past 12 months and not on life-time experiences with medical errors, limiting recall bias. We did not examine mental disorders such as major depression and post-traumatic stress disorder that could have enriched out study. Our study was aimed at medical errors and not on the broader category of adverse medical events that include events caused by medical errors or mistakes.^26^

Nevertheless, to the best of our knowledge this is the first prospective comparative population-based study among individuals affected by medical errors and the problems they face. Our findings indicate that care for victims of medical errors should screening for and target problems regarding post-event mental health, work, religion, finance and legal issues and not only address physical or medical problems.

## Supplementary data

Supplementary data are available at *EURPUB* online.

## Funding

Data of this study were extracted from the longitudinal VICTIMS study, granted by the Victims Support Foundation (Fonds Slachtofferhulp), Grant number 50006/VICTIMS, The Hague, The Netherlands.

*Conflicts of interest*: None declared.

**Key points**

- Medical errors are common but remarkably very few studies focused on the problem’s victims face.
- A prospective comparative population-based study was conducted to assess problems.
- Pre- and post-event problems were assessed varying from mental health to legal problems.
- Victims were more at risk for various post-event problems than non-affected controls.
- Care for victims of medical errors should target mental health to legal problems.

## Supplementary Material

ckaa106_supplementary_data
