# Supplementary material for: Victims of medical errors and the problems they face: a prospective comparative study among the Dutch population
Source: Eur J Public Health. 2020 Jul 10;30(6):1062–6. doi: 10.1093/eurpub/ckaa106 (PMC7733042; doi:10.1093/eurpub/ckaa106)
Supplement: ckaa106_supplementary_data [file ckaa106_supplementary_data.docx]

**Appendix 1 Results stepwise logistic regression analyses**

|  |  |  |  |  |  |  |  |
| --- | --- | --- | --- | --- | --- | --- | --- |
| Predicting Mental health problems T2 | Victims and controls | | | | | | |
|  | Adjusted OR |  | ( 95% CI) | | |  | *P* Value |
| Age |  |  |  |  |  |  |  |
| - 35-49 | 1.04 | ( | 0.69 | - | 1.57 | ) | 0.866 |
| - 50-64 | 0.71 | ( | 0.46 | - | 1.09 | ) | 0.122 |
| - ≥ 65 | 0.33 | ( | 0.20 | - | 0.56 | ) | 0.000 |
| Employments status (unemployed) | 0.56 | ( | 0.40 | - | 0.80 | ) | 0.001 |
| Physical problems (yes) | 1.69 | ( | 1.21 | - | 2.36 | ) | 0.002 |
| Mental health problems (yes) | 30.82 | ( | 22.40 | - | 42.42 | ) | 0.000 |
| Victims medical error (yes) | 3.04 | ( | 1.50 | - | 6.16 | ) | 0.002 |
|  |  |  |  |  |  |  |  |
|  |  |  |  |  |  |  |  |
| Predicting Problems with work T2 | Victims and controls | | | | | | |
|  | Adjusted OR |  | ( 95% CI) | | |  | *P* Value |
| Highest education level |  |  |  |  |  |  |  |
| - Higher secondary education^3^ | 0.32 | ( | 0.16 | - | 0.63 | ) | 0.001 |
| - Intermediate vocational^4^ | 0.73 | ( | 0.33 | - | 1.60 | ) | 0.433 |
| - Higher vocational^5^ | 0.59 | ( | 0.36 | - | 0.96 | ) | 0.034 |
| - University | 0.81 | ( | 0.52 | - | 1.25 | ) | 0.336 |
| Physical problems (yes) | 1.52 | ( | 1.04 | - | 2.21 | ) | 0.030 |
| Problems with religion (yes) | 2.74 | ( | 1.26 | - | 5.95 | ) | 0.011 |
| Problems with work (yes) | 4.63 | ( | 3.02 | - | 7.10 | ) | 0.000 |
| Victims medical error (yes) | 2.38 | ( | 1.12 | - | 5.05 | ) | 0.024 |
|  |  |  |  |  |  |  |  |
|  |  |  |  |  |  |  |  |
| Predicting Financial problems T2 | Victims and controls | | | | | | |
|  | Adjusted OR |  | ( 95% CI) | | |  | *P* Value |
| Age |  |  |  |  |  |  |  |
| - 35-49 | 1.34 | ( | 0.82 | - | 2.19 | ) | 0.248 |
| - 50-64 | 1.01 | ( | 0.62 | - | 1.66 | ) | 0.964 |
| - ≥ 65 | 0.39 | ( | 0.22 | - | 0.71 | ) | 0.002 |
| Employments status (unemployed) | 0.48 | ( | 0.32 | - | 0.71 | ) | 0.000 |
| Marital status (unmarried) | 0.71 | ( | 0.48 | - | 1.05 | ) | 0.086 |
| Financial problems (yes) | 36.85 | ( | 25.67 | - | 52.91 | ) | 0.000 |
| Victims medical error (yes) | 4.82 | ( | 2.40 | - | 9.70 | ) | 0.000 |
|  |  |  |  |  |  |  |  |

|  |  |  |  |  |  |  |  |
| --- | --- | --- | --- | --- | --- | --- | --- |
|  |  |  |  |  |  |  |  |
| Predicting Problems with religion T2 | Victims and controls | | | | | | |
|  | Adjusted OR |  | ( 95% CI) | | |  | *P* Value |
| Problems with religion (yes) | 35.01 | ( | 16.71 | - | 73.35 | ) | 0.000 |
| Victims medical error (yes) | 12.08 | ( | 5.10 | - | 28.64 | ) | 0.000 |
|  |  |  |  |  |  |  |  |
|  |  |  |  |  |  |  |  |
|  |  |  |  |  |  |  |  |
| Predicting Problems with family/partner T2 | Victims and controls | | | | | | |
|  | Adjusted OR |  | ( 95% CI) | | |  | *P* Value |
| Highest education level |  |  |  |  |  |  |  |
| - Higher secondary education^3^ | 0.31 | ( | 0.19 | - | 0.52 | ) | 0.000 |
| - Intermediate vocational^4^ | 0.50 | ( | 0.24 | - | 1.02 | ) | 0.057 |
| - Higher vocational^5^ | 0.47 | ( | 0.29 | - | 0.76 | ) | 0.002 |
| - University | 0.68 | ( | 0.44 | - | 1.05 | ) | 0.080 |
| Physical problems (yes) | 1.43 | ( | 1.01 | - | 2.01 | ) | 0.042 |
| Problems with family/partner (yes) | 11.93 | ( | 8.33 | - | 17.09 | ) | 0.000 |
| Financial problems (yes) | 2.73 | ( | 1.76 | - | 4.22 | ) | 0.000 |
|  |  |  |  |  |  |  |  |
|  |  |  |  |  |  |  |  |
|  |  |  |  |  |  |  |  |
| Predicting Legal / administrative problems T2 | Victims and controls | | | | | | |
|  | Adjusted OR |  | ( 95% CI) | | |  | *P* Value |
| Mental health problems (yes) | 2.93 | ( | 1.71 | - | 5.03 | ) | 0.000 |
| Problems with family/partner (yes) | 0.42 | ( | 0.19 | - | 0.96 | ) | 0.038 |
| Financial problems (yes) | 3.43 | ( | 1.87 | - | 6.28 | ) | 0.000 |
| Legal/ administrative problems (yes) | 8.67 | ( | 4.79 | - | 15.70 | ) | 0.000 |
| Victims medical error (yes) | 4.00 | ( | 1.80 | - | 8.89 | ) | 0.001 |
|  |  |  |  |  |  |  |  |

|  |  |  |  |  |  |  |  |
| --- | --- | --- | --- | --- | --- | --- | --- |
|  |  |  |  |  |  |  |  |
| Predicting ‘Any’ problem T2 | Victims and controls | | | | | | |
|  | Adjusted OR |  | ( 95% CI) | | |  | *P* Value |
| Age |  |  |  |  |  |  |  |
| - 35-49 | 1.10 | ( | 0.80 | - | 1.51 | ) | 0.552 |
| - 50-64 | 0.82 | ( | 0.60 | - | 1.12 | ) | 0.210 |
| - ≥ 65 | 0.46 | ( | 0.32 | - | 0.65 | ) | 0.000 |
| Gender (females) | 1.28 | ( | 1.02 | - | 1.60 | ) | 0.035 |
| Employments status (unemployed) | 0.59 | ( | 0.45 | - | 0.76 | ) | 0.000 |
| Physical problems (yes) | 0.76 | ( | 0.60 | - | 0.98 | ) | 0.032 |
| Any problem (yes) | 16.30 | ( | 12.92 | - | 20.56 | ) | 0.000 |
| Victims medical error (yes) | 5.08 | ( | 2.86 | - | 9.02 | ) | 0.000 |
|  |  |  |  |  |  |  |  |
|  |  |  |  |  |  |  |  |
|  |  |  |  |  |  |  |  |
| Predicting Depression and anxiety symptoms T2 | Victims and controls | | | | | | |
|  | Adjusted OR |  | ( 95% CI) | | |  | *P* Value |
| Age |  |  |  |  |  |  |  |
| - 35-49 | 0.85 | ( | 0.61 | - | 1.18 | ) | 0.339 |
| - 50-64 | 0.49 | ( | 0.35 | - | 0.70 | ) | 0.000 |
| - ≥ 65 | 0.30 | ( | 0.20 | - | 0.44 | ) | 0.000 |
| Employments status (unemployed) | 0.59 | ( | 0.45 | - | 0.77 | ) | 0.000 |
| Physical problems (yes) | 1.46 | ( | 1.12 | - | 1.91 | ) | 0.006 |
| Mental health problems (yes) | 12.00 | ( | 9.33 | - | 15.45 | ) | 0.000 |
| Problems with religion (yes) | 3.19 | ( | 1.70 | - | 5.97 | ) | 0.000 |
| Problems with family/partner (yes) | 1.48 | ( | 1.00 | - | 2.20 | ) | 0.050 |
| Victims medical error (yes) | 2.16 | ( | 1.19 | - | 3.94 | ) | 0.012 |
|  |  |  |  |  |  |  |  |
|  |  |  |  |  |  |  |  |
| Predicting Physical problems T2 | Victims and controls | | | | | | |
|  | Adjusted OR |  | ( 95% CI) | | |  | *P* Value |
| Age |  |  |  |  |  |  |  |
| - 35-49 | 1.70 | ( | 1.23 | - | 2.35 | ) | 0.001 |
| - 50-64 | 2.74 | ( | 2.02 | - | 3.71 | ) | 0.000 |
| - ≥ 65 | 2.95 | ( | 2.12 | - | 4.10 | ) | 0.000 |
| Gender (females) | 1.31 | ( | 1.07 | - | 1.61 | ) | 0.009 |
| Employments status (unemployed) | 0.73 | ( | 0.57 | - | 0.94 | ) | 0.014 |
| Physical problems (yes) | 15.34 | ( | 12.49 | - | 18.83 | ) | 0.000 |
| Mental health problems (yes) | 2.02 | ( | 1.47 | - | 2.78 | ) | 0.000 |
| Victims medical error (yes) | 4.93 | ( | 2.64 | - | 9.21 | ) | 0.000 |
